# Norwegian midwives’ opinion of their midwifery education – a mixed methods study

**DOI:** 10.1186/s12909-017-0917-0

**Published:** 2017-05-03

**Authors:** Mirjam Lukasse, Anne Marie Lilleengen, Anne Margrethe Fylkesnes, Lena Henriksen

**Affiliations:** 1 Department of Nursing and Health Promotion, Faculty of Health Sciences, Oslo and Akershus University College of Applied Science, PB 4 St. Olavs plass, N-0130 Oslo, Norway; 2 Division of General Gynaecology and Obstetrics, Oslo University Hospital, Postboks 4950, Nydalen, 4 St. Olavs plass, N-0130 Oslo, Norway

**Keywords:** Professionalization, Transition, Internship, Clinical practice, Midwifery identity

## Abstract

**Background:**

Midwifery education in Norway has undergone radical reforms in the past few decades. In 2004, the compulsory year of paid internship was removed from the requirement to become an authorised midwife. Since then, authorisation as a midwife depends on the successful completion of a two-year full-time academic course, consisting of 50% clinical practice and 50% theoretical education. Our objective was to examine midwives’ opinion of their Norwegian midwifery education in relation to their midwifery practice, comparing those educated with internship to those without.

**Methods:**

We performed a mixed-methods study based on data from a nationwide cross-sectional survey. A sample of 547 midwives completed a postal questionnaire, autumn 2014. Midwives were asked how they were educated, how their education prepared them for practice (multiple choice) and to freely comment on their midwifery education. Thematic analysis and descriptive and comparative analysis was used. Data sets were analysed independently and jointly interpreted.

**Results:**

Of our sample, 154 (28.2%) were educated through a two-year midwifery education without internship, while 393 (71.8%) had a one-year midwifery education with internship. Compared to midwives who had internship, midwives without were four times more likely to report that their education did not, or only partially prepare them for their work as a midwife. The association lost its significance when adjusted for experience as a midwife. According to the qualitative data, the primary reason for the association was insufficient clinical practice during education. Relevant clinical placement, ample practice time with good preceptorship and internship were proposed as methods to prepare for practice as a midwife. The theory–practice gap was highlighted as another hindrance to being prepared for practice.

**Conclusions:**

Academisation of the midwifery education has resulted in newly qualified midwives feeling less prepared for practice. Midwives would have liked more time for clinical practice and simulation training of core midwifery clinical skills included in the education. Midwifery educations need to explore ways to achieve a good balance between practice and theory. Workplaces need to explore alternative ways to internship to assist new graduates to become confident midwives with a strong midwifery identity.

**Electronic supplementary material:**

The online version of this article (doi:10.1186/s12909-017-0917-0) contains supplementary material, which is available to authorized users.

## Background

The aim of midwifery education is to prepare students to enter the profession of midwifery with the required competencies to provide safe and high standard evidence based maternity care for women experiencing complex pregnancies and births while also supporting normal pregnancy and birth [1, 2]. The International Confederation of Midwives has advocated a global standard for midwifery education [3]. Yet midwifery education varies considerably across countries [4]. Midwifery education is influenced by society and women’s needs and the context midwives work in [1, 2, 5]. The majority of midwives in Norway work in obstetrician led maternity units. Midwives share antenatal and reproductive health care in the community with general practitioners. Norway has few midwifery-led units; few independent midwives and planned home-births are rare. Nevertheless, midwives experience a great deal of autonomy within the given structure while women-centred care has limited focus [6, 7].

### Recent history of midwifery education in Norway

In Norway, midwifery education became a “specialisation in nursing” in 1952, when being a qualified nurse became a requirement for entering into midwifery education [8]. The length of the midwifery education was 18 months. In 1969, a period of internship was added to the education [8]. In 1980, the midwifery education moved from being hospital-based to school-based. It now took two full calendar, not academic, years to become an authorised midwife. The first 12 months at college consisted of a combination of 40% theory and 60% practice. The next 12 months of internship at the hospital consisted of 100% practice [9]. Midwifery students and interns had no more than 5 weeks holidays as is custom for employees in Norway. The midwifery thesis was written during the first three to 4 months of the internship [9]. During the internship intern midwives received an income and were under the jurisdiction of the Norwegian Directorate of Health and no longer under the Norwegian Directorate [9]. How much paid leave interns were given to write their thesis was at the discretion of their employer. Some interns had to write their thesis entirely in their spare time.

In 2004, new legislation determined that the midwifery education would be a two-year full-time education after nursing and the period of internship was removed [10, 11]. From 2004, all theory and practice (50:50 ratio) became included in two academic years of 40 weeks each [12]. This posed a dramatic change, as the amount of practice was reduced from 60 to 50% in the first year and from 100 to 50% in the second year, in addition to less weeks of education due to long academic holidays.

The Bologna process of harmonisation of higher education in Europe has led to an increased implementation of a common degree structure and qualification framework, also in nursing [13]. As midwifery already was a two-year post-graduate education (after the BSc in nursing), one of the midwifery organisations and several midwifery schools started to work towards converting the postgraduate course into a master level degree in midwifery [14]. Norway has five midwifery educations in total, placed at either a University college or University. Since 2012, three of these have upgraded their midwifery education from a postgraduate course at diploma level to a masters education. Moving from postgraduate diploma level to masters level has had no impact on the time spend in clinical practice, which has remained at the same 50:50 ratio since 2004. The impact of the masters level is on the theoretical part which has an increased focus on research both in relation to the thesis and evidence based practice. The first midwives qualifying with a masters in midwifery in Norway did so in 2014 and none are included in this study.

The removal of the year of internship and increasing focus on academic content in the midwifery education has caused concern that midwives at the point of registration (authorisation) are not competent and confident practitioners. Two Norwegian studies have examined how students and midwives educated after 2004 experienced their educational program [15, 16]. The most recent study reported that while the program was rated well, newly qualified midwives did not feel competent to manage complicated situations [15]. Internationally, a number of studies report that the transition from student midwife to newly qualified midwife is challenging and suggest that the educational program influences how prepared midwives feel for practice [17–20]. There are no studies in Norway comparing the opinion on the midwifery educational program of midwives educated with a year of internship to midwives without a year of internship.

### Aim/objectives

The overall aim of our study is to investigate midwives’ opinion of their Norwegian midwifery education in relation to their midwifery practice in Norway. Firstly, we examined how well midwives felt prepared for midwifery practice and compared those with and without a year of internship. Secondly, we analysed the responses to the open-ended question on midwifery education to add insight in midwives’ opinion of their midwifery education.

## Methods

### Design, sample and data-collection

In our cross-sectional nation-wide survey, we collected both quantitative and qualitative data allowing a mixed methods approach to our research question. September 2014, questionnaires, together with a response envelope, were sent to a random sample of 1500 midwives registered with either one of the two midwifery unions in Norway. We approached the unions as their members are the most likely ones to be practicing midwives. Unions also have some information on this due to varying membership rates according to practicing status. While the Norwegian Directorate of Health has a register of all persons with the authorization to practice as a midwife in Norway, they have no information if the persons authorized actually practice as a midwife. In Norway it is extremely rare for midwives not to be a member of a union, so we estimate that only a handful of active midwives were not included in the population from which the sample was drawn. The sample invited comprised approximately half of those working in midwifery in Norway. The printer performed the random sampling and posting of the questionnaires. No reminder was sent as the questionnaire was anonymous and the researchers knew neither whom they were sent to, nor who responded. The e-mail address of the main investigator was included in the questionnaire for queries.

Of the 1500 midwives, 1458 were eligible after exclusion of 26 due to wrong address, and 16 who no longer worked in midwifery. Of the 1458 eligible, 598 completed the questionnaire, 41%. For this particular study we removed from our dataset midwives who reported having had a direct-entry midwifery education (18, all educated outside of Norway) and those who indicated having had yet another type of education (33, most of them educated outside of Norway and a few part of a special educational project in Bergen in the early nineties). Thus the sample of this study was 547 midwives, 393 who had a one-year midwifery education with internship and 154 midwives who had completed a two-year midwifery without internship.

The midwives were asked information about their age (in categories), civil status, level of education, number of years of work experience as a midwife, main working area and size of the unit they currently were working at (categorized, in number of birth a year). The survey included a question asking midwives how they were educated to become midwives with the answering options: one-year course after nursing followed by one-year of internship, two-year university college course without internship, direct entry (without nursing first), other. Midwives were so categorised into two groups for analyses, those educated with internship and those without internship. Midwives with other types of education were removed from the sample as described earlier. Midwives’ were asked how they experienced that their midwifery education prepared them for working as a midwife. This question has the answering options, very well, well, partially and not at all. The options “partially” and “not at all” were recoded into not feeling prepared for the regression analysis.

Finally, midwives were invited to comment what in their opinion had been good or lacking in their midwifery education. They had a third of an A4 page to write a response. In addition, midwives could write comments on the final page. The responses to the open-ended question were marked with the same groups as used in the quantitative analysis, those with and without a year of internship. To allow some quantitative analyses the comments were coded into four categories: negative comments, positive comments, a combination of both or neutral and no or irrelevant comments.

### Ethics

The study was performed in accordance to Declaration of Helsinki. The study was submitted to the Medical and Health Research Ethics board of Southern Norway, who deemed their approval was not required as the study was not within their scope (Ref. 2014/153/REK Sør-Øst). The Norwegian Social Science Services (NSD) approved the study (Ref 38,201/3/IB). Participants were informed that a returned and completed questionnaire was considered consent.

### Analyses of the open-ended question

**Fig. 1:**
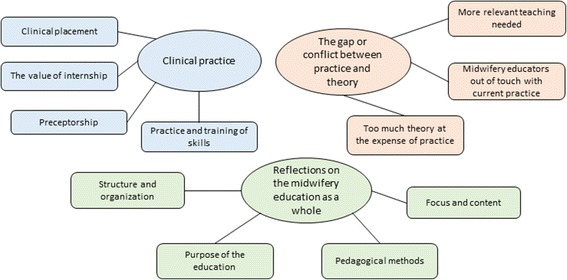
Final thematic map of themes and subthemes

The 6 phases of thematic analyses as presented by Braun and Clarke [21] were used to analyse the qualitative data. Phase one consist of becoming familiar with the data. To achieve this, the first author wrote all the comments into a word table, in Norwegian. All four authors received a copy of this document and read all the comments. Phase 2 is about generating initial codes. The first author systematically worked through the pages of comments, assessing each comment for meaning regarding our research question. Text units relating to the same topic were coded as such. A new word document was created and the codes were written in the column beside the comment they were derived from. Comments were coded as belonging to several potential themes when appropriate. The initial codes were examined and discussed by all authors. Phase 3: searching for themes and sub-themes. An initial thematic map was drawn, including 5 main themes with sub-themes (Additional file 1). This initial thematic map was evaluated and discussed by all four authors before proceeding to phase 4 which is the reviewing of themes. In phase 4 the first author reread each one of the extracts of text (comments) belonging to each of themes to check if they formed a coherent meaning. For each of the extracts of text not fitting well into the themes it was considered if a new theme or sub-theme needed to be created or if the extract should be discarded as it was of little relevance for the study. In level two of phase 4 all the authors considered the validity of the themes in relation to the entire data set and if they accurately reflected the meaning evident in the data set. This led to a reduction from 5 to 3 main themes. At this stage, the comments of those with and without internship were separated in individual files and the comments for each group read by group to see if they differed by the type of comments provided. Once more the first author read the entire set of comments to ascertain they matched the themes well and to re-code those that did not. Finally, the four authors agreed on the names and definition of the remaining themes and sub-themes (phase 5) and a final thematic map was drawn (Fig. 1). Phase 6 the production of results and discussion are to be found in their respective sections.

### Statistical analysis

Cross-tabulation and Pearson’ chi-squared test were used to study percentages and assess differences in demographic and work related factors for midwives educated with and without a year of internship. Cross-tabulation and Pearson chi-squared test were also used to compare midwives educated with a year of internship with those without internship on how they felt their education prepared them for practice and the type of comments they provided. Binary logistic regression analysis was performed to investigate the relationship between type of midwifery education and not feeling prepared to work as a midwife. This association was adjusted for age, size of the unit midwives worked at in model 1. Number of years of experience as a midwife was added to these in model 2. All analyses were two sided at α = 0.05. Statistical package SPSS version 22 was used to conduct all analyses.

### Data integration

Integration of both quantitative and qualitative data is cited as the essence of mixed methods research. [22]. Both data sets were analysed concurrently, independently of each other. Qualitative data was used to gain additional information on the quantitative results and to achieve a deeper understanding of midwives’ opinion of their midwifery education.

## Results

### Quantitative data

Of our sample, 154 (28.2%) had a two-year midwifery education without internship while 393 (71.8%) had a one-year midwifery education with internship. Midwives who had internship were significantly older and had more years of experience (Table 1). Midwives without internship more often had a bachelor degree (*P* < 0.001). Midwives with internship worked more often in community antenatal care, Out Patient Department (OPD) and in education and management, while those without internship were more often working on labour, postnatal or combined labour/postnatal wards at larger maternity units (Table 1). Of all the midwives around 8 % were currently studying and over half of them worked part-time (Table 1).

**Table 1:** Characteristics by type of midwifery education after completed nursing education

	One year midwifery education with internship *n* = 393	Two year midwifery education without internship *n* = 154	Total *N* = 547	X^2^ test
	*n* (%)	*n* (%)	*n* (%)	*P*-value
Age				
25–29	0	12 (7.8)	12 (2.2)	<0.001
30–34	0	64 (41.6)	64 (11.7)	
35–39	24 (6.1)	39 (25.3)	63 (11.5)	
40–49	105 (26.7)	35 (22.7)	140 (25.6)	
50–59	192 (48.9)	4 (2.6)	196 (35.8)	
≥ 60	72 (18.3)	0	72 (13.2)	
Civil status				
Married/co-habiting	334 (85.0)	136 (88.3)	470 (85.9)	0.315
Not married/co-habiting	59 (15.0)	18 (11.7)	77 (14.1)	
Main area of work				
Community A/N care	95 (24.2)	12 (7.8)	107 (19.6)	<0.001
Labour	90 (22.9)	55 (35.7)	145 (26.5)	
Out-patients Department	34 (8.7)	5 (3.7)	39 (7.1)	
Postnatal ward	17 (4.3)	9 (5.8)	26 (4.8)	
Combined labour & postnatal	99 (25.2)	64 (41.6)	163 (29.8)	
Midwifery led unit	14 (3.6)	5 (3.2)	19 (3.5)	
Education/Management	21 (5.3)	2 (1.3)	23 (4.2)	
Other	23 (5.9)	2 (1.3)	25 (4.6)	
Years worked as a midwife				
< 1 year	0	12 (7.8)	12 (2.2)	<0.001
1 to 2 years	1 (0.3)	21 (13.6)	22 (4.0)	
≥ 2 to 5 years	1 (0.3)	50 (32.5)	51 (9.3)	
≥ 5 to 10 years	20 (5.1)	67 (43.5)	87 (15.9)	
≥ 10 to 20 years	162 (41.2)	2 (1.3)	164 (30.0)	
≥ 20 years	209 (53.2)	0	209 (38.2)	
Missing	0	2 (1.3)	2 (0.4)	
Highest level of education achieved				
Bachelor	101 (25.7)	123 (79.9)	224 (41.0)	<0.001
Masters	28 (7.1)	9 (5.8)	37 (6.8)	0.592
Studying now	26 (6.6)	18 (11.7)	44 (8.0)	0.050
Current job				
Fulltime	185 (47.1)	62 (40.3)	247 (45.2)	0.092
Part-time	201 (51.1)	88 (57.1)	289 (52.8)	
Bank/Pool	3 (0.8)	4 (2.6)	7 (1.3)	
Missing	4 (1.0)	0	4 (0.7)	
Number of births at unit				
Not applicable	21 (18.3)	6 (3.9)	78 (14.3)	<0.001
< 500	57 (14.5)	19 (12.3)	76 (13.9)	
500–1500	74 (18.8)	23 (14.9)	97 (17.7)	
≥ 1500–2500	58 (14.8)	27 (17.5)	85 (15.5)	
≥ 2500–4000	53 (13.5)	32 (20.8)	85 (15.5)	
≥ 4000	79 (20.1)	46 (29.9)	125 (22.9)	
Missing	0	1 (0.6)	1 (0.2)	

Compared to midwives with internship those without internship more often reported that their education did not or only partially prepared for their work as a midwife (*P* < 0.001) (Table 2).

**Table 2:** Evaluation of midwifery education, comparing types of education*

	One year midwifery education with internship *n* = 393	Two year midwifery education without internship *n* = 154	Total *N* = 547
How did the midwifery education prepare you for your work as a midwife?	*n* (%)	*n* (%)	*n* (%)
Very well	168 (42.7)	25 (16.2)	189 (35.3)
Well	190 (48.3)	82 (53.2)	269 (49.8)
Partially	35 (8.9)	44 (28.6)	79 (14.5)
Not at all	0	2 (1.3)	2 (0.4)
Missing	0	1 (0.6)	1 (0.2)

*P < 0.001 chi-squared test

Midwives without internship were four times more likely to report not feeling prepared for working as a midwife, crude OR 4.39, 95% CI (2.69–7.18) (not in the tables). This association remained significant after adjusting for age and working at a large maternity unit, AOR 3.84, 95% CI (1.80–8.20) (not in the tables). The association between being educated without internship and not feeling prepared for work disappeared after additionally adjusting for years of experience as a midwife, AOR 3.24 (0.91–11.52) (not in the tables).

Of 393 midwives with internship 155 (39.4%) provided comments on their education and 90 (58.4%) out of 154 midwives without internship. Midwives without internship were significantly (*P* < 0.001) more likely to have negative comments (Table 3).

**Table 3:** Comments on midwifery education, comparing types of education*

	One year midwifery education with internship *n* = 393	Two year midwifery education without internship *n* = 154	Total *N* = 547
	*n* = (%)	*n* = (%)	*n* = (%)
Negative comments	55 (14.0)	58 (37.7)	113 (20.7)
Positive comments	61 (15.5)	10 (6.5)	71 (13.0)
Both negative and positive or neutral comments	39 (9.9)	22 (14.3)	61 (11.2)
No or irrelevant comments	238 (60.6)	64 (41.6)	302 (55.2)

*P < 0.001 chi-squared test

### Qualitative data

Except for a few exceptions, the comments were mostly short and to the point, varying from one to five lines. Data analysis revealed three overarching themes: 1) Clinical practice, 2) The gap or conflict between practice and theory, and 3) reflections on the midwifery education as a whole. Midwives educated with internship more often commented on the importance of clinical practice while midwives without internship had more comments on the gap between theory and practice and the midwifery education as a whole. The themes are presented in Table 4, with sub-themes and supporting comments, and are described below.

**Table 4:** Themes, sub-themes and supporting comments from midwives (I = midwives educated with internship, N = midwives educated without internship)

Themes	Sub-themes	Supporting comments
Clinical practice	Practice and training of skills	“*Missed more simulation training using a model or 3-D computer program for suturing, delivering, supporting the perineum, emergencies*” #133 (I)
		“*wanted more simulation training on emergencies*” #26 (N)
	Clinical placement	“*Good with practice at a midwifery-led unit, a smaller and large unit. Sad for midwifery students who risk being at one hospital for all their practice*” #19 (I)
		“*Reflecting now I think the school should have made a clinical placement at a large hospital with more pathology compulsory*.” #122 (N)
	The value of internship	“*It was fantastic the internship. Feel that today’s newly qualified midwives are not prepared and fearful to start working after 2 years at school”* #56 (I)
		“*would have liked to have had internship – safe, gradual transition*” #20 (N)
	Preceptorship	“*the level of theoretical knowledge varied too much among contact-midwives (preceptors)*” #6 (I)
		“*Better follow-up of students who have experienced tough situations… Better supervision from teachers from school in practice*” #465 (N)
The gap or conflict between practice and theory	Too much theory at the expense of practice	“*There should be more practice and less focus on theory and written assignments. Ours is a practical profession, and I think it* *now* *drowns in theory…*” #36 (I)
		“*Lots of research, this at the expense of other things that were more important.”* #15 (N)
	More relevant teaching	“*Focus was on normal childbirth. Poorly prepared for technology, complications and emergency situations”* #353 (N)
		*“Very much focus on psycho-social relations in the education. This is important but when you work as a midwife in a high-tech birth clinic you need concrete knowledge.”* #64 (I)
	Midwifery educators out of touch with current practice	“*less good, teachers who were removed from practice, they knew too little about everything that happens in a labour room*” #8 (I)
		“*Missed having educators/teachers at the school who kept themselves up to date by working in a maternity unit”* #20 (N)
Reflections on the midwifery education as a whole	Structure and organization	“*Have by now met many newly qualified Danish midwives. They radiate real professional confidence which is so unusual among our newly qualified colleagues. Have real faith in direct entry midwifery, where there is time and space for midwifery professionalism building identity*” # 416 (I) “*Want direct entry*” #24 (N)
		“*I think it is a real strength to have nursing as a base. See this in particular when we now have Danish temporary staff who lack this. They lack something, much they are unable to do because they lack nursing”* # 598 (I)
	Purpose of the education	“…*education helped to establish the basis for my view on childbirth which in its origin is a normal process which women have mastered for 10.000 years. Without this basis I could have ended up as an obstetric nurse (also because I had to become a nurse first)”* #24 (N)
		“*Research is good but of those who complete midwifery are there few who become researchers*” # 15 (N)
		“*We were educated to be independently thinking midwives ….”* # 39 (I)
	Pedagogical methods	“*felt the education was self-study*” #475 (N)
		“*Fantastic wonderful study with inspiring teachers and lecturers*” #62 (I)
		“*the education was very good, up to date on midwifery, new articles and literature*” #24 (N)
	Focus and content	“*Missed proper focus on the midwife’s role and responsibility in normal labour, how a midwife can promote natural birth in collaboration with a childbearing woman.”* # 73 (I)
		“*Too much focus on analyses + writing of good articles, too little teaching in practical midwifery*” #61 (N)

### Theme 1. Clinical practice

Midwives with and without internship alike emphasized that midwifery is a practical profession and learning core midwifery clinical skills is paramount to midwifery education.

We identified four sub-themes.

#### Practice and training of skills

Many midwives expressed the need for more clinical practice and more simulation training of basic midwifery skills and obstetrics emergencies. In particular, midwives with internship conveyed their appreciation of having had lots of clinical practice. For example, participant # 63 “*Good with lots of practice!*” While midwives without internship more often commented that they had wanted more clinical practice. As participant # 127 commented “*Too little practice. The transition to starting work at the labour ward was too big*”.

#### Clinical placement

Most midwives seemed to be of the opinion that having varied clinical placements was advantageous. The type of placement influences future midwifery attitudes. As participant # 339 wrote *“…had clinical placements at all levels of birthing units, inclusive midwifery-led unit, which was very important for my own attitude towards normal birth.*” Many midwives, in particular those with internship, expressed the lack of or too little clinical placement in community antenatal care and reproductive health care. As for example participant #376 “*No preparation with practice to become a community midwife*.”

#### The value of internship

Fifty-four of the midwives educated with internship commented on the value of internship. Only four of these were negative about their internship. For all four of them lack of mentorship was an issue. As participant #68 wrote “*Lacked mentorship during my internship. Difficult situations had to be handled alone several times. Unsafe*.” All the other midwives expressed strong appreciation for their internship and stated how it gave them sorely needed experience and made them feel confident. Participant #75 wrote, “*Am of the opinion that the year of internship gave me confidence to tackle the challenges I meet*.” A few midwives educated without internship explicitly wrote about the benefits of internship. Participant #377 wrote “*Sadly too little practice – would have benefited from having internship*…”.

#### Preceptorship

Midwives with and without internship alike expressed the pivotal role preceptorship in clinical practice plays in creating a good learning environment and promoting learning. Those who had experienced good or satisfactory supervision mentioned this in very few words. As participant #298 wrote “*Had good, direct preceptors*”. However, there were many negative comments, describing unkind treatment of students, poor quality of supervision, lack of continuity and too little follow-up by midwifery educators from college. As illustrated by participant #99’s comment, “*Some places did not treat their students in a respectful way – difficult to keep ones confidence and feel safe performing tasks.”*

Theme 2. The gap or conflict between theory and practice.

Both midwives educated with and without internship commented the gap or conflict they experienced between what is taught and prioritized in midwifery education and what is practiced and valued in clinical practice. We categorized these comments in three sub-themes.

#### Too much theory at the expense of clinical practice

Midwives without internship expressed concern that in their education; too much time was spent on theory and research at the expense of gaining practical experience. As exemplified by participant #119 “*Felt that lots of scientific theory took “the space” from practical experience something clinical supervisors also pointed out”.* Midwives with internship did not express this concern about their own education but about the education without internship. For example participant #36 commented “*Experience that today’s students have too much focus on research and that the education is losing “clinical acumen”/volume of deliveries.”*

#### More relevant teaching needed

Midwives frequently mentioned that teaching in college focused on normal childbirth while in practice they met pathology. For example, participant #84 wrote “*Good with large focus on normality! But not a satisfactory preparation for practice in a large maternity unit with a lot of pathology…”* Time in teaching was spent on topics which in practice seemed of little use or relevance. As commented by participant #137 “…*more teaching on CTG and less weight on research and theory for which I have had little use in “the real midwife’s life”.”* According to the midwives, teaching did not meet the challenges midwives experience in every day practice.

#### Midwifery educators out of touch with current practice

Many midwives were negative about midwifery educators who had not been in clinical practice for many years and who were not up to date with current clinical practice. Both those with and without internship alike mentioned this explicitly and implied this when commenting on bad quality teaching by midwifery educators as opposed to good teaching by visiting lecturers from clinical practice. Teaching which was out of date caused either a gap or conflict between teaching and practice. For example participant #149 “*Taught by midwives without clinical experience or long time ago since clinical practice. A lot of out of date teaching. Most of the learning had to be fixed in practice – almost only there.”*

Theme 3. Reflections on the midwifery education as a whole.

Midwives made comments on their own education and others’. This theme showed a great variety and conflicting opinions on the midwifery education. We identified 4 sub-themes.

#### Structure and organization

Midwives were positive about the structure of modules, i.e. weeks of theory followed by weeks of practice. For example participant # 38 “*Good with periods of theory and then to be in practice for several weeks at the same place.*” Midwives educated with internship were critical of the degree system while midwives without internship were of the opinion they should have had a master degree. For example participant #36 without internship wrote “… *and I feel that all these “degrees” bachelor/master* etc. *are unnecessary*.” Compared to participant #161 without internship who commented “*Missed that the midwifery education was/is not a master*.” Some midwives were negative about having to become a nurse first, while others commented that it was helpful being a nurse first (Table 4). Midwives’ opinions were divided about written assignments during clinical placement. For example participant #334 wrote “*…too much self-study/written assignments during practice periods. At times this gave 70-80 hours of work a week”.* Compared to participant #42 “*practice and written assignments together, that was good”.* It is important that the theoretical tasks given during clinical practice are relevant to the particular placement. As participant #31 commented “*Theoretical part and there it was placed in practice did not mach. One learns better if one experiences what one should learn in theory.”*

#### The purpose of midwifery education

The midwifery education is expected to qualify midwives who are confident, competent in clinical practice, can be independent and who have a clear midwifery identity. For example participant #318 “*Felt that we so long ago received a great midwifery identity and were interested in alternative births.”* Midwifery identity included focus on and promotion of normal birth. Participant #17 wrote “*Good with focus on normal birth …need that ballast to work for midwifery”.* Being able to find evidence for practice after being qualified was considered a positive effect of the newer education. However, several midwives both with and without internship pointed out that most midwives once qualified will not become researchers.

#### Pedagogical methods

Midwives educated without internship had twice as many comments about pedagogical methods than midwives educated with internship. Midwives appreciated lecturers from clinical practice and wanted more inspiring lecturers. Midwives were positive about up to date literature and content; and negative when this was not the case. Both midwives with and without internship were negative about the amount of self-study in the midwifery education. Participant # 324 wrote *“Too much self-study and Problem Based Learning in the theoretical part in my opinion.”* Independent of type of education did midwives mention too many written assignments as well as wanting less group work, more cases, more brainstorming and most of all more practice on phantoms, i.e. simulation training also in college.

#### Content and focus

Midwives with and without internship wrote down topics on which they would have wanted more teaching on in college. Topics mentioned most often were antenatal care, pain management in labour, postpartum care, breastfeeding, reproductive health, and observation of the newborn. Furthermore, midwives wanted to learn how to teach women coping strategies, coaching and motivational interviewing. Independent of type of education did midwives mention that they missed focus on midwifery itself, the midwife’s role and responsibility. Participant #101 wrote “*The basis year could have been even more related to fundamental midwifery*”. Midwives with one-year education with internship wished they had learned about evidence based practice. Participant #69 “*At the time I did not miss it, but now I miss having had an introduction to evidence based practice and method.”*

## Discussion

A third of the midwives educated after 2004 did “*not*” or “*only partially*” feel prepared to work as a midwife. The primary reason for not feeling prepared for practice according to the qualitative results is insufficient clinical practice. The midwifery education in Norway has a 50/50 ratio of practice and theory and requires students to have “performed” 50 spontaneous vaginal births, 10 more than required by the EU directives [23]. It seems the workforce in Norway expects newly qualified midwives to be just as competent and confident as previously newly authorised midwives were, i.e. those who had completed a year of internship. However, the year of internship was not part of the midwifery education. The year of internship was in fact a compulsory transition program, required by the Department of Health before authorisation. The extensive body of research on graduate nurse transition programs shows that these programs emerged to facilitate and support the development and integration of new graduates [24, 25]. Common elements of these transition programs are formal or informal preceptorship, mentorship and supernumerary time [24, 25]. In our study, most midwives who had an internship described this as a positive learning experience that gave them skills and confidence. They describe their status as protected, safe and mentored. This is in sharp contrast to the study by Hamre which reported that most intern midwives almost immediately became part of the ordinary staff without preceptorship and little assistance unless they specifically asked for it [9]. The question remains if the expectations of knowledge, skills and confidence, by the workforce and newly qualified midwives themselves, are realistic. The literature suggests that the transition from student to graduate unavoidably is a challenge [15, 17, 19, 20, 26]. That the most important requirement for a midwife at registration is to practice safely [27]. Furthermore, newly qualified midwives’ new level of responsibility stimulates an awareness of the importance of continuous professional education (CPE) [19, 26, 27]. While a compulsory year of internship is unlikely to be implemented again, appropriate supportive programs for newly qualified midwives with a named preceptor should be explored.

Our participants pointed out that relevant clinical placements, ample of practice time and good preceptors were pivotal to learning midwifery competencies and gaining a midwifery identity. There is extensive evidence supporting the importance of good preceptorship in midwifery [28–30].

The second reason for midwives not feeling prepared for practice, suggested by the qualitative data, is the gap or even conflict between what is taught at the midwifery program and what is practiced in the clinical environment. The comments from the midwives are supported by the literature, which shows that midwifery education teaches “women-centred care”, “believing in a woman’s ability to give birth”, “birth as a natural process” and “birth as a whole process” [26, 31]. These philosophical stances and approaches are in contrast to the medical model of care found in most institutions where midwives work [26, 31]. The transition of midwifery students to graduate midwives involves therefore how to deal with the chasm in belief systems and to develop their own unique professional midwifery identity negotiating environmental and organisational constraints [26, 31].

The reform in the midwifery education in Norway in 2005 added a stronger focus on academic content to the program. The academisation has continued as more midwifery educations in Norway either have become or are seeking to become master courses. The new courses aim at better equipping midwives to access and critically analyse relevant research in order to meet the demands of modern maternity care, to practice evidence based, to influence the care of women on an equal footing with other health professionals and to develop the midwifery profession [4, 32]. Many respondents were critical to the focus on research and written assignments in the midwifery education. This raises the question if the midwifery educations in Norway have failed to make obvious the relevance and need for research in midwifery. Our findings are similar to a Swedish study which asked students, midwives and obstetricians how well the midwifery education prepared for clinical practice [18].

The participants in our study were negative about the midwifery educators who were not up to date with current clinical practice. In Europe, and Norway, evidence suggests that nursing and midwifery educators are increasingly required to be in the possession of a doctoral degree for appointment as a lecturer [4, 33]. Once in education, midwifery educators are expected to meet higher education institutions’ expectations as researchers and develop pedagogical expertise [34]. Thus, midwifery educators are stimulated to become academic scholars while practical skill instruction appears to be less valued [33]. In contrast to the ICM recommended standards, midwifery educators in Norway are not obliged to maintain competence in midwifery clinical practice once they have been appointed at an educational institution [35]. It seems that becoming a lecturer inevitable leads to midwifery educators becoming more distant to clinical practice [34]. It seems reasonable to consider that these midwifery educators make valuable contributions to theoretical teaching and midwifery research and that preceptors, who are mostly in clinical practice and guest lecturers can ensure adequate teaching of clinical skills and current clinical practice [29].

Midwives had strong but conflicting opinions about the organisation, structure and purpose of the midwifery program. The long-standing debate among midwives in Norway about the pros and cons of direct-entry midwifery education was reflected in the responses of the participants. Direct-entry midwifery education is currently not an option in Norway and local and national political interests are not in favour of this option. However, the results of our study show that the current educational system, of post nursing midwifery education produces midwives who do not feel competent to manage midwifery complications and emergencies. One can argue that 3 years of nursing education contributes minimally to the required skills and competencies a newly qualified midwife needs. While one can imagine that a 5-year direct entry education could produce confident and competent midwives at the time of qualification, with a clear midwifery identity.

### Strengths and limitations

A strength of our study is the mixed methods design. While the quantitative data clearly showed significant difference in the opinion on the midwifery education between those with and without internship, the qualitative data gave insight and a deeper understanding into why these opinions differed [22]. The answers to the open-ended question were short and as such could not provide the level of rich data that individual or focus group interviews could have provided. Our method did not allow follow-up questions on expressed opinions. However, the answers were generally clear and concise, packed with meaning with few examples or illustrations. Complete anonymity may have made the participants more free to be honest in their comments. Many comments supporting the same themes was a strength. We choose thematic analysis because it is a relatively straightforward form of qualitative analysis as it is suitable for our data and does not required the same detailed theoretical and technical knowledge as other more advance methods of qualitative analysis [21]. To establish rigour and trustworthiness of the qualitative data-analysis all of the authors were closely involved in the analysis and we present and document a detailed record of the analytic process in the Additional files.

A limitation of our study is recall bias. In particular, the midwives educated with internship may have forgotten the negative things about the education and internship or the feeling of being a newly qualified and unexperienced midwife. It is difficult to assess how the response rate of 41%, a clear limitation of our study may affect the results. There is no single register of active midwives in Norway. All midwives who receive their authorization to work as a midwife are registered at the Norwegian Directorate of Health. However, they have no information if those registered practice midwifery. It is therefore not possible to compare our sample to all active midwives in Norway and evaluate how representative our sample is. Nevertheless, our sample of nearly 600 midwives includes a large proportion of the active midwives in Norway and the findings are thus likely to be representative of midwives educated in Norway.

## Conclusion and implications for practice

Midwives, both those educated with internship and those without were critical of the increased academic focus in the midwifery education. In particular, midwives educated after the 2005 reform would have like more time for clinical practice and simulation training of core midwifery clinical skills. Midwifery educations need to explore ways to achieve a good balance between practice and theory. Increased digital learning, using short videos to demonstrate clinical skills, a more extensive use of simulation training and increased availability of simulation laboratories may be some of the ways of doing so. Midwifery educations may have to find ways to integrate the research component better into the midwifery program, making it relevant to midwifery. This suggests that research methodology courses together with students without a healthcare background may not be optimal. Using midwifery examples of research while teaching methodology could prove an effective way of integrating research better into the midwifery education. Our study further suggests that midwifery leaders in the workplace need to explore ways in which newly qualified midwives can be assisted in their transition from students. Practice in other countries and research from related professions suggests that formal mentorship can be an effective and inexpensive way of going so [24].

## Additional file

Additional file 1: Initial thematic map with themes and subthemes (TIFF 162 kb)

## Abbreviations

AOR: Adjusted Odds Ratio
CI: Confidence Interval
CPE: Continuous Professional Education
Dnj: Den norske jordmorforening
NSF: Norsk Sykepleier Forbund
OPD: Out-patients department
OR: Odds Ratio
