# Supplementary figures and images for: Norwegian midwives’ opinion of their midwifery education – a mixed methods study
Source: BMC Med Educ. 2017 May 3;17:80. doi: 10.1186/s12909-017-0917-0 (PMC5415717; doi:10.1186/s12909-017-0917-0)

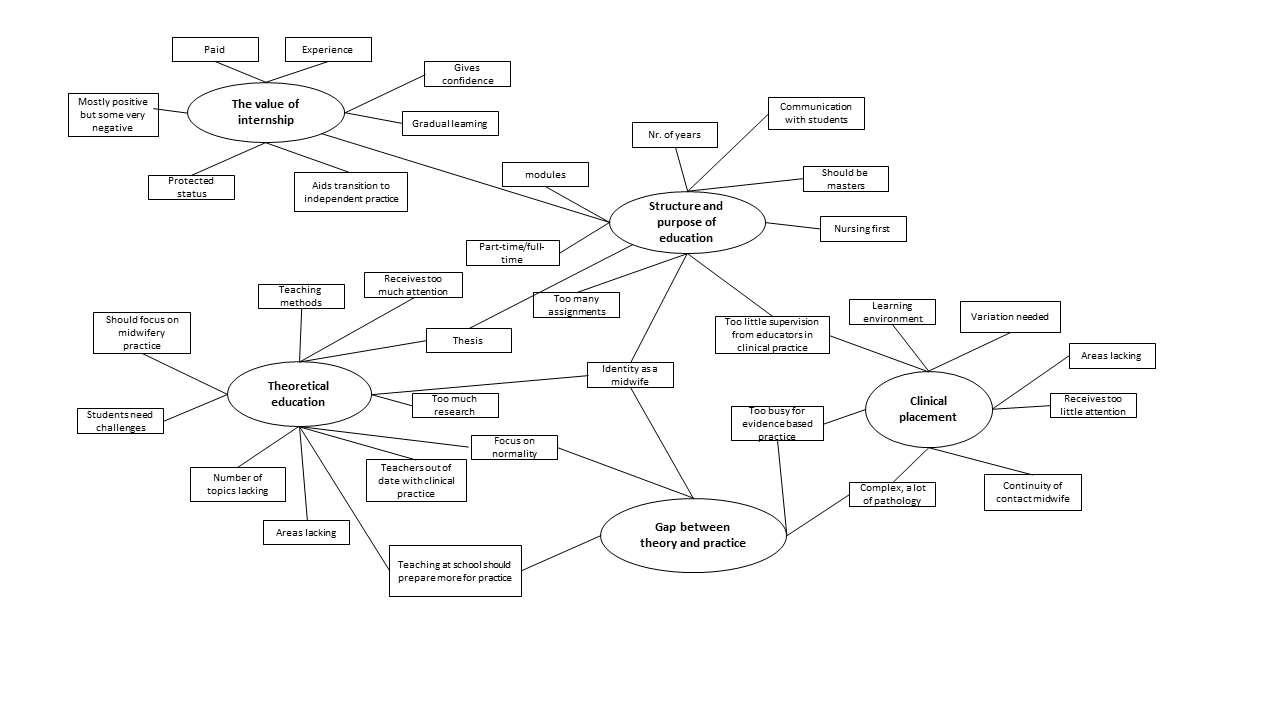

Supplement: Additional file 1: — Initial thematic map with themes and subthemes (TIFF 162 kb) [file 12909_2017_917_MOESM1_ESM.tif]
